# Supplementary material for: Identification of Alternative Mitochondrial Electron Transport Pathway Components in Chickpea Indicates a Differential Response to Salinity Stress between Cultivars
Source: Int J Mol Sci. 2020 May 28;21(11):3844. doi: 10.3390/ijms21113844 (PMC7312301; doi:10.3390/ijms21113844)
Supplement: Supplementary file 1 [file ijms-21-03844-s001.pdf]

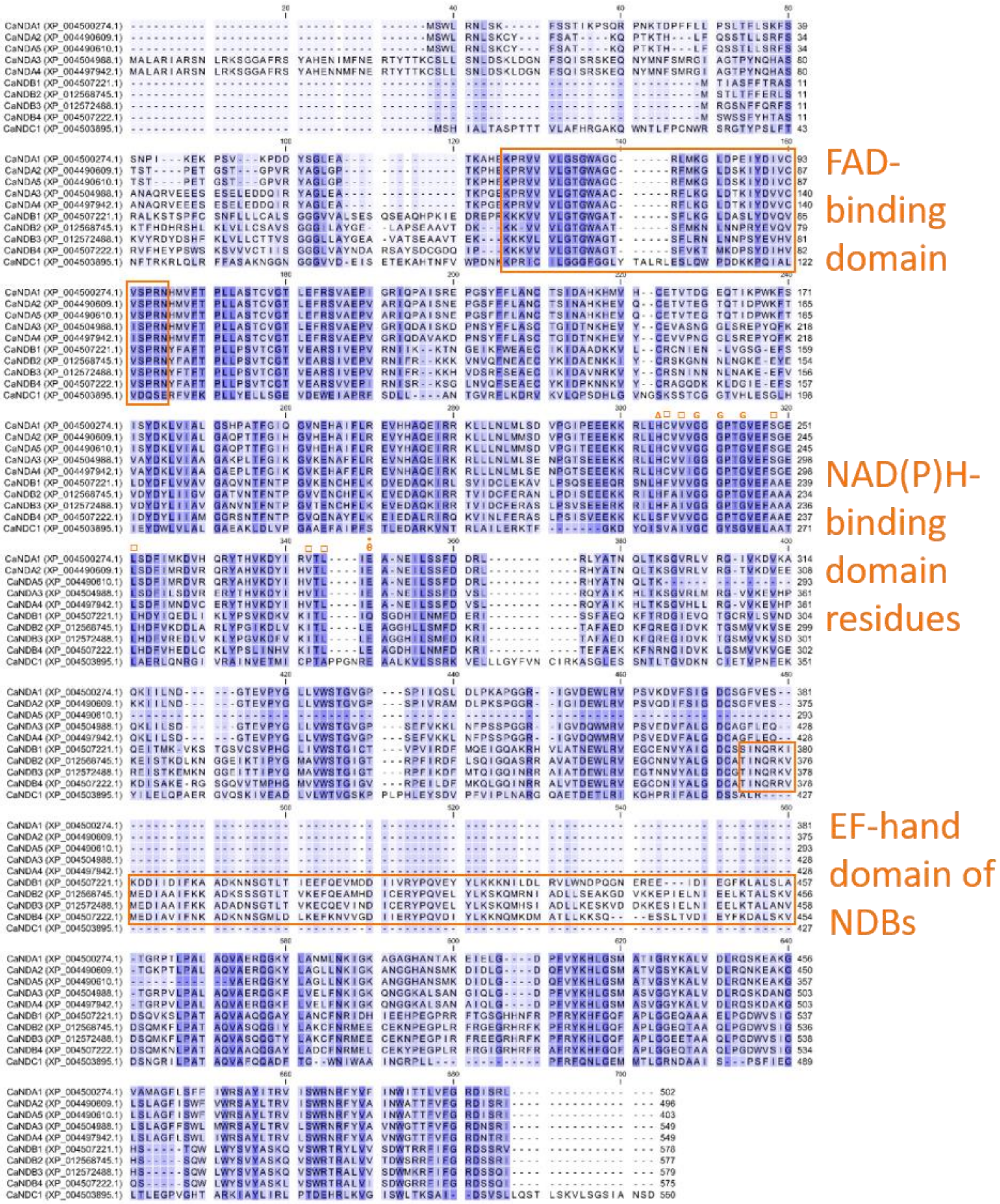

Figure S1: Chickpea ND protein alignments indicating FAD-binding domain, substrate-binding domain and EF-hand domain. Alignment generated using CLC Sequence Viewer v8 (Qiagen Aarhus A/S). The FAD-binding domain, and the EF-hand domain that is characteristic of NDB (Michalecka et al. 2003) are indicated by orange boxes. Residues are highlighted within the NAD(P)H-binding domain; Δ, basic or hydrophilic; □, hydrophobic; θ, acidic; and G, Glycine. The acidic residue thought to dictate specificity for NADH or NADPH is indicated with an asterisk; glutamate (E) for NADH and typically either glutamine (Q) or asparagine (N) residues for NADPH (Geisler et al. 2007; Hao & Rasmusson 2016).

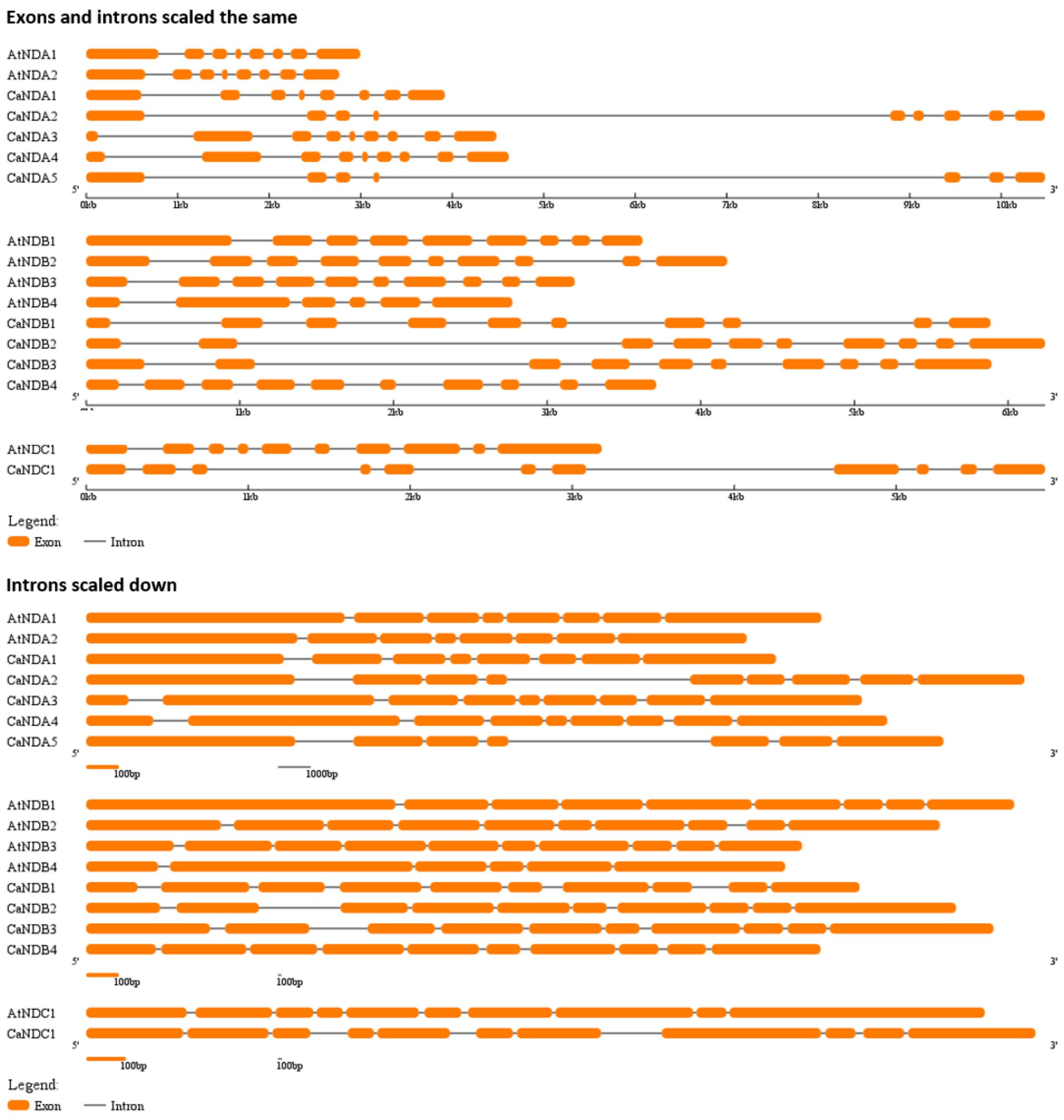

Figure S2: Chickpea and Arabidopsis ND gene structure comparisons. Shown with continuous scales (top) or with introns scaled down for clearer comparison of exons (bottom). Images generated using the Gene structure Display Server (http://gsds.cbi.pku.edu.cn/).

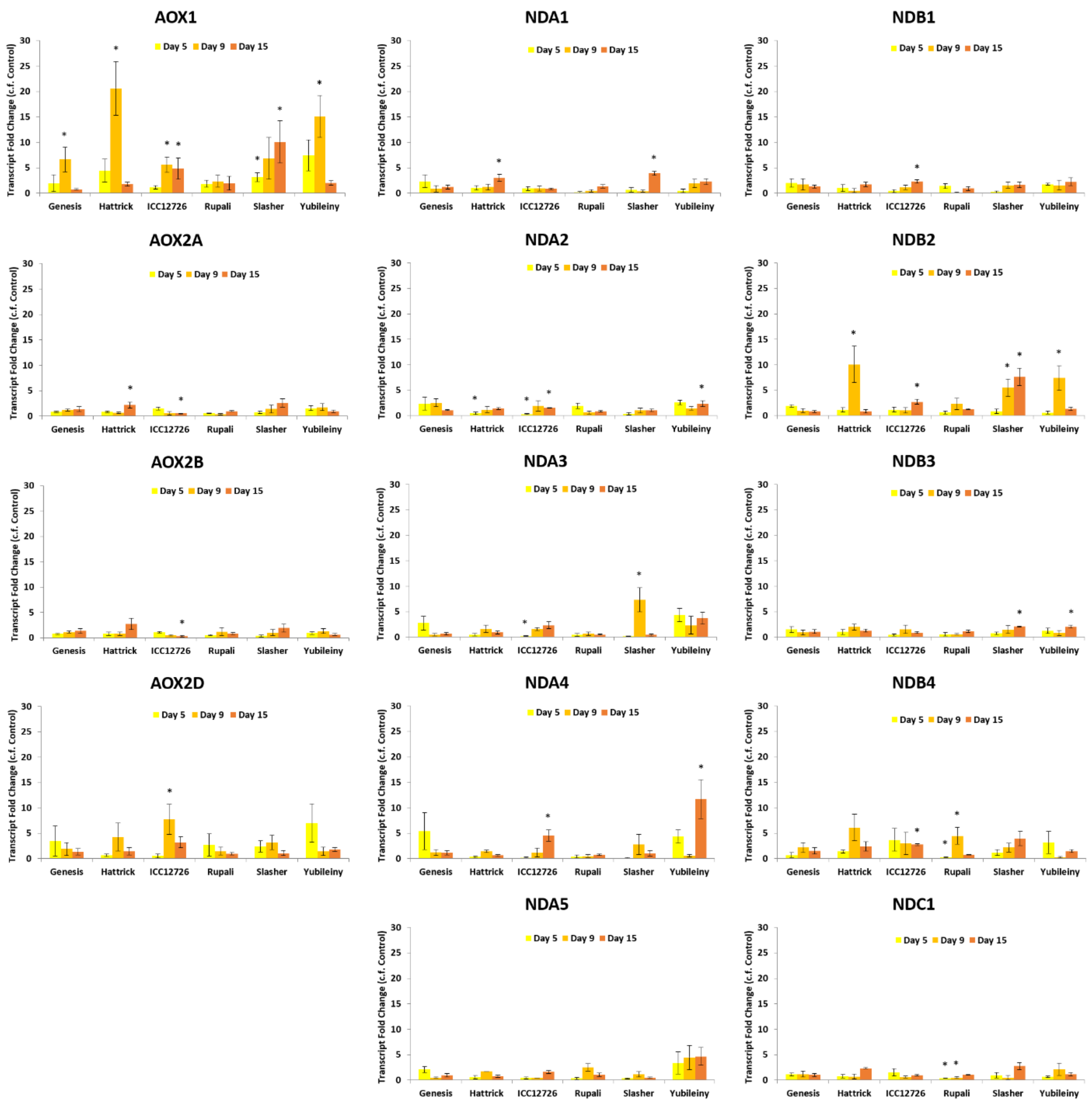

Figure S3: Time-dependent response of AOX and NAD(P)H DH genes to salinity stress. Expressed as fold change relative to control samples. Statistical significance indicated by * (p<0.05) based on individual Mann-Whitney U tests between control and salt-treated samples for each gene, time point and cultivar. (n = 4 ± S.E.M.)

Gene Ortholog	Cajanus cajan (LIS)		Phaseolus vulgaris (LIS)		Vignus unguiculata (LIS)		Glycine max (LIS)		Glycine max (GV)		Medicago truncatula (GV)		Lotus japonicus (LjGEA)	
	Leaf/shoot	Root	Leaf/shoot	Root	Leaf/shoot	Root	Leaf/shoot	Root	Leaf/shoot	Root	Leaf/shoot	Root	Leaf/shoot	Root
NDA1	23	0.1	47	0.1	8	0.1	20<br>9	0<br>0	13	8	14	8	200	7
NDA2	12<br>23	28<br>0.2	0.9<br>1.9	17<br>6	11	31	1<br>8	7<br>3	13	14	14	14	3164	2274
NDA3	0.1	3	0.1	5	2	0.6	0.9<br>0	1<br>0.1	10	11	-	-	60	463
NDA4	-	-	-	-	-	-	-	-	-	-	-	-	-	-
NDB1	8	10	5	62	12	9	14	10	13	13	13	13	1582	963
NDB2	0.1	1	0	0.3	0.6	1.4	0.6<br>0	3<br>0.1	-	-	-	-	72	1062
NDB3	2.5	12	13	4	11	34	4<br>6	8<br>6	15	15	13	13	3795	4551
NDB4	0	0	-	-	0	0	0.2<br>0	0<br>3	-	-	-	-	0	0.3
NDC1	10	5	13	4	15	2	21	1	14	10	15	10	951	676

Figure S4: ND expression in shoot (or leaf) vs root tissues of other legumes. Data presented as FPKM from RNAseq transcriptomic datasets, sourced from Legume Information System (LIS) and *Lotus japonicus* Gene Expression Atlas (LjGEA) or as normalized expression values sourced from Genevestigator (GV).

A

		AOX1	AOX2A	AOX2B	AOX2D	NDA1	NDA2	NDA3	NDA4	NDA5	NDB1	NDB2	NDB3	NDB4	NDC1
AOX1	Spearman Coefficient	1.000	0.657	-0.029	0.257	0.543	0.086	0.600	0.543	0.314	0.200	0.771	0.486	-0.086	0.429
	Sig. (2-tailed)		0.156	0.957	0.623	0.266	0.872	0.208	0.266	0.544	0.704	0.072	0.329	0.872	0.397
AOX2A	Spearman Coefficient		1.000	0.371	-0.086	0.257	0.257	0.600	0.371	0.257	0.714	0.314	-0.029	-0.771	0.429
	Sig. (2-tailed)			0.468	0.872	0.623	0.623	0.208	0.468	0.623	0.111	0.544	0.957	0.072	0.397
AOX2B	Spearman Coefficient			1.000	-.943**	0.086	-0.200	-0.029	-0.543	0.771	0.086	0.086	-.886*	-0.543	0.371
	Sig. (2-tailed)				0.005	0.872	0.704	0.957	0.266	0.072	0.872	0.872	0.019	0.266	0.468
AOX2D	Spearman Coefficient				1.000	0.143	0.371	0.200	0.600	-0.714	0.086	0.029	.943**	0.314	-0.143
	Sig. (2-tailed)					0.787	0.468	0.704	0.208	0.111	0.872	0.957	0.005	0.544	0.787
NDA1	Spearman Coefficient					1.000	0.429	0.086	-0.257	0.314	-0.143	0.429	0.200	-0.143	.829*
	Sig. (2-tailed)						0.397	0.872	0.623	0.544	0.787	0.397	0.704	0.787	0.042
NDA2	Spearman Coefficient						1.000	-0.371	0.086	-0.543	0.600	-0.486	0.257	-0.486	0.657
	Sig. (2-tailed)							0.468	0.872	0.266	0.208	0.329	0.623	0.329	0.156
NDA3	Spearman Coefficient							1.000	0.429	0.314	0.086	0.714	0.257	-0.143	-0.200
	Sig. (2-tailed)								0.397	0.544	0.872	0.111	0.623	0.787	0.704
NDA4	Spearman Coefficient								1.000	-0.429	0.486	0.200	0.714	0.086	-0.257
	Sig. (2-tailed)									0.397	0.329	0.704	0.111	0.872	0.623
NDA5	Spearman Coefficient									1.000	-0.371	0.657	-0.543	-0.086	0.257
	Sig. (2-tailed)										0.468	0.156	0.266	0.872	0.623
NDB1	Spearman Coefficient										1.000	-0.371	0.029	-0.771	0.257
	Sig. (2-tailed)											0.468	0.957	0.072	0.623
NDB2	Spearman Coefficient											1.000	0.257	0.257	0.086
	Sig. (2-tailed)												0.623	0.623	0.872
NDB3	Spearman Coefficient												1.000	0.429	-0.086
	Sig. (2-tailed)													0.397	0.872
NDB4	Spearman Coefficient													1.000	-0.486
	Sig. (2-tailed)														0.329
NDC1	Spearman Coefficient														1.000
	Sig. (2-tailed)														

0.8 > R2 > 0.7 and p < 0.1
0.9 > R2 > 0.8 and p < 0.05
R2 > 0.9 and p < 0.001

B

		AOX1	AOX2A	AOX2B	AOX2D	NDA1	NDA2	NDA3	NDA4	NDA5	NDB1	NDB2	NDB3	NDB4	NDC1
AOX1	Spearman Coefficient	1.000	-0.029	-0.314	0.143	0.257	0.086	-0.086	0.314	0.029	0.429	.829*	0.371	0.543	0.257
	Sig. (2-tailed)		0.957	0.544	0.787	0.623	0.872	0.872	0.544	0.957	0.397	0.042	0.468	0.266	0.623
AOX2A	Spearman Coefficient		1.000	.886*	-0.371	.829*	-0.257	-0.486	-0.429	-.829*	-0.257	0.257	0.714	0.429	.829*
	Sig. (2-tailed)			0.019	0.468	0.042	0.623	0.329	0.397	0.042	0.623	0.623	0.111	0.397	0.042
AOX2B	Spearman Coefficient			1.000	-0.486	0.714	-0.429	-0.543	-0.771	-.886*	-0.429	0.086	0.486	0.257	0.714
	Sig. (2-tailed)				0.329	0.111	0.397	0.266	0.072	0.019	0.397	0.872	0.329	0.623	0.111
AOX2D	Spearman Coefficient				1.000	-0.371	.943**	.886*	0.600	0.543	.943**	0.257	-0.314	0.257	-0.371
	Sig. (2-tailed)					0.468	0.005	0.019	0.208	0.266	0.005	0.623	0.544	0.623	0.468
NDA1	Spearman Coefficient					1.000	-0.200	-0.371	-0.429	-0.600	-0.143	0.429	.943**	0.257	1.000**
	Sig. (2-tailed)						0.704	0.468	0.397	0.208	0.787	0.397	0.005	0.623	
NDA2	Spearman Coefficient						1.000	.943**	0.657	0.600	.886*	0.143	-0.086	0.086	-0.200
	Sig. (2-tailed)							0.005	0.156	0.208	0.019	0.787	0.872	0.872	0.704
NDA3	Spearman Coefficient							1.000	0.600	0.771	0.771	-0.086	-0.257	-0.200	-0.371
	Sig. (2-tailed)								0.208	0.072	0.072	0.872	0.623	0.704	0.468
NDA4	Spearman Coefficient								1.000	0.714	0.543	-0.029	-0.143	-0.029	-0.429
	Sig. (2-tailed)									0.111	0.266	0.957	0.787	0.957	0.397
NDA5	Spearman Coefficient									1.000	0.429	-0.314	-0.371	-0.543	-0.600
	Sig. (2-tailed)										0.397	0.544	0.468	0.266	0.208
NDB1	Spearman Coefficient										1.000	0.543	-0.086	0.429	-0.143
	Sig. (2-tailed)											0.266	0.872	0.397	0.787
NDB2	Spearman Coefficient											1.000	0.371	.829*	0.429
	Sig. (2-tailed)												0.468	0.042	0.397
NDB3	Spearman Coefficient												1.000	0.143	.943**
	Sig. (2-tailed)													0.787	0.005
NDB4	Spearman Coefficient													1.000	0.257
	Sig. (2-tailed)														0.623
NDC1	Spearman Coefficient														1.000
	Sig. (2-tailed)														

0.8 > R2 > 0.7 and p < 0.1
0.9 > R2 > 0.8 and p < 0.05
R2 > 0.9 and p < 0.001

Figure S5: Correlation coefficients between AP genes in response to salinity stress. Salt-induced expression changes (fold change) were used in Spearman correlation analyses to identify co-expression patterns of AP genes in the salinity response at (A) day 9 of salt treatment and (B) day 15 of salt treatment.. Significant relationships are highlighted according to the key * p<0.05, ** p<0.01.

			CaAOX1			CaNDB2			CaAOX2A		
Site Name	function	sequence	Position	Strand	Matrix score.	Position	Strand	Matrix score.	Position	Strand	Matrix score.
	From Thirkettle-Watts et al. (2003)										
SEF1-binding site	may be involved in young tissue development	ATATTTA(A/T)(A/T)	10	–	N/A						
SEF1-binding site	may be involved in young tissue development	ATATTTA(A/T)(A/T)	93	+	N/A	83	+	N/A			
SEF1-binding site	may be involved in young tissue development	ATATTTA(A/T)(A/T)	566	+	N/A						
SEF1-binding site	may be involved in young tissue development	ATATTTA(A/T)(A/T)	871	+	N/A						
SEF1-binding site	may be involved in young tissue development	ATATTTA(A/T)(A/T)	1032	+	N/A						
SEF1-binding site	may be involved in young tissue development	ATATTTA(A/T)(A/T)	1078	-	N/A	1707	-	N/A			
SEF1-binding site	may be involved in young tissue development	ATATTTA(A/T)(A/T)	1647	+	N/A						
SEF1-binding site	may be involved in young tissue development	ATATTTA(A/T)(A/T)	1856	+	N/A						
S1F box	cis-acting negative element in non-photosynthetic tissue	ATGGTA	1639	+	N/A						
I Box,	cis-acting element involved in light-responsiveness	GATAAG	1933	+	N/A						
ASF-1 motif	TGA1a-binding site, high root expression	TGACG				1331	+	N/A	485	–	N/A
	From Clifton et al. (2005)										
GT1 CORE		TCGATA	680	+	N/A	1310	+	N/A	1528	–	N/A
MYB plant		AAACCGGA	643	–	N/A						
W Box	part of a light responsive element	GGTCAA				1892	–	N/A			
G Box	cis-acting regulatory element involved in light responsiveness	CACGTG	401	+	N/A						
–		GCGTGT				1875	–	N/A			
	From PlantCARE										
AAGAA-motif		GAAAGAA							849	+	7
AAGAA-motif		gGTAAAGAAA	2001	-	7	1518	-	9	913	-	9
ABRE	cis-acting element involved in the abscisic acid responsiveness	ACGTG	400	+	6	882	–	5	167	–	5
ABRE	cis-acting element involved in the abscisic acid responsiveness	ACGTG	401	+	5	1677	–	5			
ABRE	cis-acting element involved in the abscisic acid responsiveness	ACGTG	1951	+	5	1989	–	5			
ABRE	cis-acting element involved in the abscisic acid responsiveness	TACGTGTC				1675	–	8			
ABRE3a		TACGTG				1677	–	6	167	–	6
ABRE4		CACGTA				1677	+	6	167	+	6
AP-1		TGAGTTAG							1398	+	8
ARE	cis-acting regulatory element essential for the anaerobic induction	AAACCA				1230	+	6			
ARE	cis-acting regulatory element essential for the anaerobic induction	AAACCA	347	-	6				760	-	6
ARE	cis-acting regulatory element essential for the anaerobic induction	AAACCA							1080	–	6
AT-rich element	binding site of AT-rich DNA binding protein (ATBP-1)	ATAGAAATCAA				715	–	10			
AT~ABRE		TACGTGTC				1675	–	8			
Box 4	part of a conserved DNA module involved in light responsiveness	ATTAAT	222	+	6	105	+	6			
Box 4	part of a conserved DNA module involved in light responsiveness	ATTAAT				370	+	6			
Box 4	part of a conserved DNA module involved in light responsiveness	ATTAAT				381	+	6			
Box 4	part of a conserved DNA module involved in light responsiveness	ATTAAT	583	+	6	555	+	6			
Box 4	part of a conserved DNA module involved in light responsiveness	ATTAAT				831	+	6			
Box 4	part of a conserved DNA module involved in light responsiveness	ATTAAT				934	+	6			
Box 4	part of a conserved DNA module involved in light responsiveness	ATTAAT	999	+	6	945	+	6			
Box 4	part of a conserved DNA module involved in light responsiveness	ATTAAT	1060	+	6	1051	+	6			
Box 4	part of a conserved DNA module involved in light responsiveness	ATTAAT	1218	–	6						
Box 4	part of a conserved DNA module involved in light responsiveness	ATTAAT	1733	–	6						
Box 4	part of a conserved DNA module involved in light responsiveness	ATTAAT	1788	-	6				1016	-	6
Box 4	part of a conserved DNA module involved in light responsiveness	ATTAAT	2106	-	6				1238	-	6
Box II	part of a light responsive element	ACACGTTGT				881	+	9			
CGTCA-motif	cis-acting regulatory element involved in the MeJA-responsiveness	CGTCA				1330	–	5	685	+	5
CGTCA-motif	cis-acting regulatory element involved in the MeJA-responsiveness	ATTTTAAA							1867	+	5
ERE		ATTTCATA				527	–	8	496	+	8
ERE		ATTTTAAA	709	+	8	923	–	8	541	+	8
ERE		ATTTTAAA	1034	-	8	1005	-	8	1272	-	8
ERE		ATTTTAAA							1440	–	8
F-box		CTATTCTCATT							1152	+	10
G-Box	cis-acting regulatory element involved in light responsiveness	CACGTT	400	+	6	882	+	6			
G-box	cis-acting regulatory element involved in light responsiveness	CACGTT	400	+	6	1989	+	6	1361	+	9
G-Box	cis-acting regulatory element involved in light responsiveness	TACGTG	1950	–	6	1677	–	6	167	–	6
GA-motif	part of a light responsive element	ATAGATAA	122	–	8						
GCN4_motif	cis-regulatory element involved in endosperm expression	TGAGTCA							886	+	7
Gap-box	part of a light responsive element	CAAATGAA(A/G)A							780	–	9.5
GT1-motif	light responsive element	GGTTAA				1614	+	6			
LAMP-element	part of a light responsive element	CTTTATCA							1261	–	8
MBS	MYB binding site involved in drought-inducibility	CAACTG				1025	–	6			
MSA-like	cis-acting element involved in cell cycle regulation	TCAAACGGT							1659	+	9
MYB		CAACCA	1699	+	6	1022	+	6	1552	+	6
MYB		CAACCA	1925	+	6						
MYB		CAACCA	2017	+	6				261	–	6
MYB		CAACCA	1370	-	6	1452	-	6	872	-	6
MYB		TAACCA	1753	-	6	1613	-	6	876	-	6
MYC		CATTTG	654	+	6	613	+	6	611	+	6
MYC		CATGTG	1842	-	6	1734	-	6	784	+	6
Myb		CAACTG	887	-	6	1025	-	6			
Myb		TAACTG				1863	–	6			
Myb-binding site		CAACAG				1022	+	6	872	–	6
STRE	stress-responsive element	AGGGG	1996	–	5				20	–	5
TC-rich repeats	cis-acting element involved in defense and stress responsiveness	ATTCTCTAAC				1464	+	9			
TCA-element	cis-acting element involved in salicylic acid responsiveness	CCATCTTTTT							1843	+	9
TCA-element	cis-acting element involved in salicylic acid responsiveness	CCATCTTTTT							273	–	9
TCA-element	cis-acting element involved in salicylic acid responsiveness	CCATCTTTTT							1073	–	9
TCA-element	cis-acting element involved in salicylic acid responsiveness	CCATCTTTTT				1445	-	9	1307	-	9
TCCC-motif	part of a light responsive element	TCTCCCT				2090	+	7			
TCT-motif	part of a light responsive element	TCTTAC	897	+	6						
TGACG-motif	cis-acting regulatory element involved in the MeJA-responsiveness	TGACG				1330	+	5	685	–	5
TGACG-motif	cis-acting regulatory element involved in the MeJA-responsiveness	TGACG							1867	–	5
Unnamed__1	60K protein binding site	GAATTTAATTAA	1061	–	11	927	+	11			
Unnamed__1	60K protein binding site	GAATTTAATTAA	1789	–	11						
Unnamed__1	60K protein binding site	GAATTTAATTAA	2103	–	11						
Unnamed__1		CGTGG	402	+	5	1988	–	5	1584	+	5
Unnamed__2		AACCTAACCT				619	+	9			
Unnamed__4		CTCC	144	+	4				599	+	4
Unnamed__4		CTCC	949	+	4				857	+	4
Unnamed__4		CTCC							863	+	4
Unnamed__4		CTCC	1285	+	4	2062	+	4	1770	+	4
Unnamed__4		CTCC	2007	+	4	2091	+	4	1837	+	4
Unnamed__4		CTCC	2064	+	4	2095	+	4	1841	+	4
Unnamed__4		CTCC				915	-	4	691	-	4
Unnamed__4		CTCC				2075	-	4	1249	-	4
WUN-motif		CAATTACAT							563	–	9
WUN-motif		AAATTACT							927	–	8
WUN-motif		AAATTACTA							926	–	9
W box	part of a light responsive element	TTGACC				1891	+	6			
as-1		TGACG				1330	+	5	685	–	5
as-1		TGACG							1867	–	5
chs-CMA1a		TTACTTAA				1341	–	8			

Figure S6: Analysis of 2kb upstream regions of stress-inducible *CaAOX1* and *CaNDB2* and 1.6 kb upstream region of non-stress-inducible *CaAOX2A*, for common cis-acting regulatory elements from Thirkettle-Watts et al (2003), Clifton et al. (2005) and Lescot et al. (2002). Yellow highlight indicates potentially matching elements in two or more upstream regions.

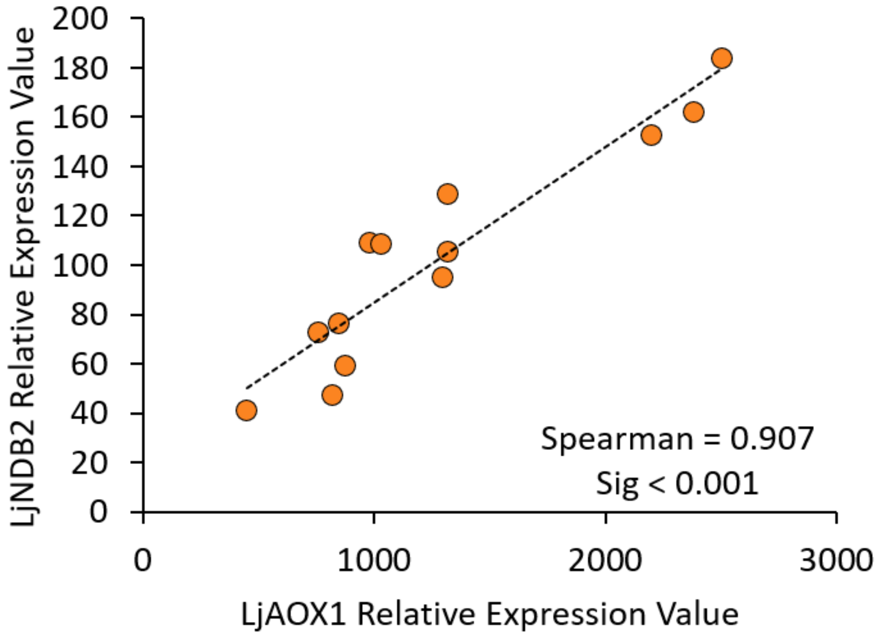

Figure S7: Co-ordinated expression of *LjAOX1* and *LjNDB2* in salinity stress experiments with *Lotus japonicus* shoot tissue. Comparisons were made based on normalized expression data from Genechip® (Affymetrix) microarray experiments, available on Lotus Base. Data represent means (6-7 biological replicates) from two studies (Sanchez et al. 2008; Sanchez et al. 2011) and were analyzed here using a Spearman correlation analysis in SPSS (v25, IBM) and plotted using Excel (Microsoft).

Table S1: Sub-cellular localization of chickpea, soybean and Arabidopsis ND proteins based on signal peptide prediction tools. Probability scores included where available. For MitoProtII and ChloroP ≥0.5 is considered significant and for Predotar ≥0.2 may be significant (Claros et al. 1996; Emanuelsson et al. 1999; Small et al. 2004).

**Protein**	**Summary**	**iPSORT**	**TargetP(2.0)**		**MitoProtII**		**ChloroP**		**Predotar**	
Chickpea										
CaNDA1	mitochondrion/chloroplast	Mit	Mit	0.70	Mit	0.53	Chl	0.54	None	
CaNDA2	mitochondrion/chloroplast	Mit	Mit	0.82	Mit	0.54	Chl	0.56	Mit	0.23
CaNDA3	mitochondrion	Mit	Mit	0.46	Mit	0.96	Non-Chl	0.47	Mit	0.61
CaNDA4	mitochondrion	Mit	Mit	0.36	Mit	0.97	Non-Chl	0.48	Mit	0.23
CaNDA5	mitochondrion/chloroplast	Mit	Mit	0.82	Mit	0.54	Chl	0.51	Mit	0.23
CaNDB1	mitochondrion/chloroplast	Mit	Mit	0.26	Mit	0.84	Chl	0.51	Mit	0.46
CaNDB2	other/mitochondrion	Mit	Other	-	Non-Mit	0.33	Non-Chl	0.44	None	-
CaNDB3	other/mitochondrion	Mit	Other	-	Non-Mit	0.3	Non-Chl	0.44	None	-
CaNDB4	other/mitochondrion	Mit	Other	-	Non-Mit	0.35	Non-Chl	0.45	None	-
CaNDC1	chloroplast/mitochondrion	Chl	Chl	0.23	Mit	0.98	Chl	0.56	Chl	0.73
Soybean										
GmNDA1.1	mitochondrion/chloroplast	Mit	Mit	0.75	Mit	0.69	Chl	0.56	Mit	0.66
GmNDA1.2	mitochondrion/chloroplast	Mit	Mit	0.85	Mit	0.87	Chl	0.54	Mit	0.41
GmNDA2.1	mitochondrion/chloroplast	Mit	Mit	0.60	Mit	0.99	Chl	0.57	Mit	0.34
GmNDA2.2	mitochondrion/chloroplast	Mit	Mit	0.57	Mit	0.97	Chl	0.56	Mit	0.26
GmNDA3.1	other/mitochondrion	Other	Other	0.11	Other	0.94	Non-Chl	0.45	Mit	0.69
GmNDA3.2	other/mitochondrion	Other	Other	0.15	Other	0.92	Non-Chl	0.46	Mit	0.56
GmNDB1	other/mitochondrion	Other	Other	0.68	Other	0.82	Non-Chl	0.48	Mit	0.58
GmNDB2.1	mitochondrion/other	Mit	Mit	0.38	Mit	0.82	Non-Chl	0.47	E.R.	0.28
GmNDB2.2	mitochondrion/other	Mit	Mit	0.38	Mit	0.82	Non-Chl	0.46	None	-
GmNDB3.1	mitochondrion/other	Mit	Mit	0.34	Mit	0.60	Non-Chl	0.47	None	-
GmNDB3.2	mitochondrion/other	Mit	Mit	0.34	Mit	0.76	Non-Chl	0.47	None	-
GmNDB4.1	other/mitochondrion	Mit	Other	-	Non-Mit	0.31	Non-Chl	0.46	None	-
GmNDB4.2	other/mitochondrion	Mit	Other	-	Mit	0.58	Non-Chl	0.46	None	-
GmNDC1	chloroplast/mitochondrion	Chl	Chl	0.46	Mit	0.99	Chl	0.57	Chl	0.45
Arabidopsis										
AtNDA1	mitochondrion/chloroplast	Chl	Mit	0.48	Mit	0.71	Chl	0.54	Mit	0.51
AtNDA2	mitochondrion/chloroplast	Chl	Mit	0.71	Mit	0.96	Chl	0.55	Mit	0.59
AtNDB1	mitochondrion	Mit	Mit	0.68	Mit	0.75	Non-Chl	0.45	Mit	0.35
AtNDB2	mitochondrion/other	Mit	Mit	0.35	Mit	0.47	Non-Chl	0.46	None	-
AtNDB3	mitochondrion/other	Mit	Other	-	Non-Mit	0.12	Non-Chl	0.47	None	-
AtNDB4	mitochondrion/other	Mit	Mit	0.34	Mit	0.63	Non-Chl	0.49	None	-
AtNDC1	chloroplast/mitochondrion	Chl	Chl	0.53	Mit	0.93	Chl	0.58	Chl	0.39

Table S2: *CaND* transcript levels in various tissues. Data presented as raw FPKM from RNAseq transcriptomic datasets. GS, Germinating seedling; YL, Young leaf; SAM, Shoot apical meristem; Stages of bud and flower development 1-4 (Garg et al. 2011)

Gene Name	Identifier	Length	Shoot	Root	Mature leaf	Flower bud	Young pod	GS	YL	SAM	Flower Bud				Flower			
											1	2	3	4	1	2	3	4
CaNDA1	TC16690	1741	80.5	0.0	31.0	4.3	11.0	7.3	9.9	8.7	8.3	7.9	7.8	8.5	8.3	7.8	8.3	8.8
CaNDA2	TC08045	1926	13.4	23.6	7.8	30.3	36.6	10.3	10.7	9.3	10.3	10.9	11.0	10.2	9.7	9.8	10.6	9.8
CaNDA2	TC21163	1176	0.0	0.0	0.0	0.0	14.6	4.2	3.7	4.4	5.2	5.3	4.3	3.7	4.3	3.9	4.3	3.6
CaNDA4	TC25299	275	0.0	0.0	0.0	0.0	0.0	4.8	4.9	6.1	4.2	4.3	4.4	4.2	2.7	2.6	3.1	2.9
CaNDA4	TC29323	218	0.0	0.0	0.0	0.0	0.0	4.4	5.1	5.6	3.9	3.7	3.1	1.8	1.1	2.4	2.8	1.1
CaNDA4	TC29520	206	0.0	0.0	0.0	0.0	0.0	5.2	6.4	6.6	5.0	5.4	5.1	4.0	3.6	3.0	3.3	2.4
CaNDB1	TC16741	1888	62.6	0.0	7.8	43.2	18.3	10.4	9.6	11.0	8.9	9.4	9.4	9.4	9.5	9.4	9.9	9.6
CaNDB2	TC13391	350	0.0	0.0	0.0	0.0	0.0	4.1	4.3	2.9	6.0	4.8	3.5	3.6	2.9	3.9	2.1	2.9
CaNDB2	TC30053	215	0.0	0.0	0.0	0.0	0.0	3.2	1.9	1.8	3.8	2.7	1.5	2.6	1.7	2.4	2.1	3.1
CaNDB3	TC01306	3579	125.2	259.5	294.3	185.9	161.1	12.1	12.8	12.2	11.3	11.7	11.9	12.1	12.6	12.4	12.5	11.7
CaNDC1	TC06937	2084	46.9	0.0	38.7	43.2	22.0	9.1	11.4	9.5	10.6	10.4	10.7	10.9	11.4	11.5	10.8	11.0

Table S3: Comparative salinity tolerance levels of six chickpea cultivars based on data from Sweetman et al. (In Press). N.S. No significant difference between control and salt-treated plants. Decreased = relative to control plants.

	Classification	Fresh Weight	Dry Weight	Visible Symptoms	Na^+/K^+
Genesis	Tolerant	N.S.	Decreased	Healthy	Low
Hattrick	Sensitive	N.S.	N.S.	Poor	High
ICC12726	Moderately Sensitive	Decreased	Decreased	Healthy	High
Rupali	Sensitive	Decreased	Decreased	Poor	Low
Slasher	Tolerant	N.S.	N.S.	Healthy	High
Yubileiny	Moderately Sensitive	Decreased	N.S.	Healthy	Low

Table S4: Summary of other legume ND gene accessions and locations

Preliminary gene name	Protein accession	gDNA accession	RNA accession	Locus accession	Chromosome and location
Glycine max					
Nda1.1	XP_003538295.1	NC_016098.2	XM_003538247.3	GLYMA_11G173700	Gm11: 18900000..18904458
Nda1.2	XP_003551266.1	NC_016105.2	XM_003551218.3	GLYMA_18G061700	Gm18: 5602197..5606420
Nda2.1 X1	XP_003519255.1	NC_016089.2	XM_003519207.3	GLYMA_02G227700	Gm2: 41445717..41451151, complement
X2	XP_006575414.1	“	XM_006575351.3	“	“
Nda2.2	XP_003544888.1	NC_016101.2	XM_003544840.3	GLYMA_14G194600	Gm14: 45965435..45970801, complement
Nda3.1 X1	XP_006583797.1	NC_016094.2	XM_006583734.2	GLYMA_07G191200	Gm7: 35867618..35873670
X2	XP_003529302.1	“	XM_003529254.3	“	“
X3	XP_006583796.1	“	XM_006583733.2	“	“
X4	XP_014633598.1	“	XM_014778112.2	“	“
Nda3.2	XP_003532554.1	NC_016095.2	XM_003532506.3	GLYMA_08G058000	Gm8: 4453562..4458821, complement
Ndb1	XP_003539893.2	NC_016099.2	XM_003539845.3	GLYMA_12G107000	Gm12: 9804176..9809441, complement
Ndb2.1 X1	XP_006594628.1	NC_016100.2	XM_006594565.2	GLYMA_13G246600	Gm13: 35533867..35539249
X2	XP_006594629.1	“	XM_006594566.2	“	“
X3	XP_006594630.1	“	XM_006594567.3	“	“
X4	XP_006594631.1	“	XM_006594568.3	“	“
Ndb2.2	XP_003547132.1	NC_016102.2	XM_003547084.3	GLYMA_15G067300	Gm15: 5129299..5134400, complement
Ndb3.1	XP_003530989.1	NC_016095.2	XM_003530941.3	GLYMA_08G059300	Gm8: 4529245..4535924
Ndb3.2	XP_003529293.1	NC_016094.2	XM_003529245.3	GLYMA_07G189900	Gm7: 35773706..35779168, complement
Ndb4.1	XP_006582334.1	NC_016093.2	XM_006582271.2	GLYMA_06G298300	Gm6: 48718698..48724430
Ndb4.2	XP_014620282.1	NC_016099.2	XM_014764796.1	GLYMA_12G106900	Gm12: 9796165..9803041, complement
Ndc1	XP_003525138.1	NC_016092.2	XM_003525090.3	LOC100795521	Gm5: 37969921..37975004
Medicago truncatula					
MtNda1	XP_013460271.1	NC_016409.2	XM_013604817.1	MTR_3g464380	Mt3: 25865131..25868399, complement
MtNda2 X1	XP_003615703.2	NC_016411.2	XM_003615655.2	MTR_5g071250	Mt5: 30215877..30222533, complement
X2	XP_013454142.1	“	XM_013598688.1	“	“
MtNda3	XP_003608194.2	NC_016410.2	XM_003608146.2	MTR_4g090580	Mt4: 35914965..35919171, complement
MtNdb1 X1	XP_013455574.1	NC_016410.2	XM_013600120.1	MTR_4g045997	Mt4: 16013268..16018920, complement
X2	XP_013455573.1	“	XM_013600119.1	“	“
MtNdb2	XP_013462476.1	NC_016408.2	XM_013607022.1	MTR_2g011730	Mt2: 2921774..2927153, complement
MtNdb3 X1	XP_003608247.1	NC_016410.2	XM_003608199.2	MTR_4g091240	Mt4: 36124694..36129496
X2	XP_013457117.1		XM_013601663.1	MTR_4g091240B	
MtNdb4	XP_013455572.1	NC_016410.2	XM_013600118.1	MTR_4g045993	Mt4: 16007976..16011675, complement
MtNdc1 X1	XP_003630359.2	NC_016414.2	XM_003630311.2	MTR_8g094730	Mt8: 39549738..39555759
X2	XP_013446902.1	“	XM_013591448.1	“	“
X3	XP_013446903.1	“	XM_013591449.1	“	“

X4	XP_013446904.1	"	XM_013591450.1	"	"
X5	XP_013446905.1	"	XM_013591451.1	"	"
Phaseolus vulgaris					
PvNda1	XP_007158703.1	NC_023758.1	XM_007158641.1	PHAVU_002G175300g	Pv 2: 32571688..32574997
PvNda2	XP_007142196.1	NC_023752.1	XM_007142134.1	PHAVU_008G260300g	Pv8: 57305919..57310068
PvNda3	XP_007159264.1	NC_023758.1	XM_007159202.1	PHAVU_002G223400g	Pv2: 38785425..38789434
PvNdb1	XP_007132630.1	NC_023749.1	XM_007132568.1	PHAVU_011G111300g	Pv11: 14478133..14487078, complement
PvNdb2	XP_007148248.1	NC_023754.1	XM_007148186.1	PHAVU_006G192400g	Pv6: 29977155..29983021
PvNdb3	XP_007159245.1	NC_023758.1	XM_007159183.1	PHAVU_002G221500g	Pv2: 38574051..38578842, complement
PvNdb4	XP_007132629.1	NC_023749.1	XM_007132567.1	PHAVU_011G111200g	Pv11: 14436083..14440465, complement
PvNdc1 X1	XP_007159881.1	NC_023758.1	XM_007159819.1	PHAVU_002G275600g	Pv2: 43976921..43981738
X2	XP_007159882.1	"	XM_007159820.1	"	"
Arachis duranensis					
Nda1 X1	XP_020991306.1	NC_029772.2	XM_021135647.1	LOC107490736	A01: 5694977..5700353, complement
X2	XP_020991298.1	"	XM_021135639.1	"	"
X3	XP_015967053.1	"	XM_016111567.2	"	"
Nda2	XP_015932007.1	NC_029778.2	XM_016076521.2	LOC107458316	A07: 23971500..23975955, complement
Nda3 X1	XP_020994622.1	NC_029774.2	XM_021138963.1	LOC107480643	A03: 118225392..118229050
X2	XP_020994624.1	"	XM_021138965.1	"	"
X3	XP_020994623.1	"	XM_021138964.1	"	"
X4	XP_015956289.1	"	XM_016100803.2	"	"
X5	XP_015956288.1	"	XM_016100802.2	"	"
Ndb1.1	XP_015953489.1	NC_029774.2	XM_016098003.2	LOC107477909	A03: 17640057..17644526, complement
Ndb1.2	XP_020984985.1	NC_029779.2	XM_021129326.1	LOC107462142	A08 30965292..30969848, complement
Ndb2	XP_015943513.1	NC_029781.2	XM_016088027.2	LOC107468689	A10: 3578628..3583066
Ndb3	XP_015956271.1	NC_029774.2	XM_016100785.2	LOC107480630	A03: 117936408..117941438, complement
Ndb4	XP_020992351.1	NC_029774.2	XM_021136692.1	LOC107477979	A03: 17634938..17639343, complement
Ndc1 X1	XP_015955821.1	NC_029774.2	XM_016100335.2	LOC107480195	A03: 110247775..110251505, complement
X2	XP_015955822.1	"	XM_016100336.2	"	"
Arachis ipaensis					
Nda1 X1	XP_016162931.1	NC_029785.2	XM_016307445.2	LOC107605512	B01: 4691080..4696621
X2	XP_020971917.1	"	XM_021116258.1	"	"
X3	XP_020971920.1	"	XM_021116261.1	"	"
Nda2 X1	XP_016166289.1	NC_029791.2	XM_016310803.2	LOC107608993	B07: 25510668..25515104, complement
X2	XP_020963174.1	"	XM_021107515.1	"	"
Nda3 X1	XP_020975090.1	NC_029787.2	XM_021119431.1	LOC107631039	B03: 119383069..119387682
X2	XP_020975091.1	"	XM_021119432.1	"	"
X3	XP_016189842.1	"	XM_016334356.2	"	"
X4	XP_016189840.1	"	XM_016334354.2	"	"

X5	XP_020975089.1	“	XM_021119430.1	“	“
Ndb1.1	XP_016188330.1	NC_029787.2	XM_016332844.2	LOC107629900	B03: 20090889..20095670, complement
Ndb1.2 X1	XP_020964460.1	NC_029792.2	XM_021108801.1	LOC107613462	B08: 8833526..8838235, complement
X2	XP_020964461.1	“	XM_021108802.1	“	“
X3	XP_016170952.1	“	XM_016315466.2	“	“
Ndb2	XP_016182539.1	NC_029794.2	XM_016327053.2	LOC107624552	B10: 5398239..5402271
Ndb3	-	-	-	-	-
Ndb4	XP_020972869.1	NC_029787.2	XM_021117210.1	LOC107629901	B03: 20082439..20088160, complement
Ndc1 X1	XP_016189696.1	NC_029787.2	XM_016334210.2	LOC107630920	B03: 111780687..111784245, complement
X2	XP_016189697.1	“	XM_016334211.2	“	“
Lupinus angustifolius					
Nda1 X1	XP_019447006.1	NC_032014.1	XM_019591461.1	LOC109350264	LG06: 23056743..23069222
X2	XP_019447007.1	“	XM_019591462.1	“	“
X3	XP_019447008.1	“	XM_019591463.1	“	“
Nda2	XP_019455721.1	NC_032017.	XM_019600176.1	LOC109356695	LG09: 19308673..19313011, complement
Nda3.1	XP_019431921.1	NW_017722427.1	XM_019576376.1	LOC109339012	Unplaced scaffold: 66466..71026
Nda3.2	XP_019431919.1	NW_017722427.1	XM_019576374.1	LOC109339011	Unplaced scaffold: 58375..63226
Ndb1	XP_019448973.1	NC_032015.1	XM_019593428.1	LOC109351807	LG07: 3364236..3370499, complement
Ndb2	XP_019418371.1	NC_032024.1	XM_019562826.1	LOC109329152	LG16: 861828..866211, complement
Ndb3	XP_019431453.1	NW_017720703.1	XM_019575908.1	LOC109338635	Unscaffolded: 68061..75163
Ndb4	XP_019448972.1	NC_032015.1	XM_019593427.1	LOC109351806	LG07: 3359445..3363374, complement
Ndc1	XP_019458586.1	NC_032009.1	XM_019603041.1	LOC109358669	LG01: 25891266..25896671, complement
Vigna angularis					
Nda1	XP_017437083.1	NC_030645.1	XM_017581594.1	LOC108343361	Va9: 17310948..17314559, complement
Nda2 X1	XP_017428034.1	NC_030643.1	XM_017572545.1	LOC108336197	Va7: 13127030..13136219
X2	XP_017428035.1	“	XM_017572546.1	“	“
Nda3 X1	XP_017425772.1	NC_030637.1	XM_017570283.1	LOC108334436	Va1: 7460516..7465391, complement
X2	XP_017425780.1	“	XM_017570291.1	“	“
Ndb1	XP_017433360.1	NC_030644.1	XM_017577871.1	LOC108340465	Va8: 19718533..19725582
Ndb2 X1	XP_017435213.1	NC_030645.1	XM_017579724.1	LOC108342023	Va9: 29822527..29828501
X2	XP_017435214.1	“	XM_017579725.1	“	“
Ndb3	XP_017421035.1	NC_030637.1	XM_017565546.1	LOC108330942	Va1: 7677535..7682130
Ndb4	-	-	-	-	-
Ndc1	XP_017412250.1	NC_030637.1	XM_017556761.1	LOC108323929	Va1: 1142346..1147308, complement
Vigna radiata					
Nda1	XP_014518625.1	NC_028360.1	XM_014663139.2	LOC106775902	Vr10: 9587937..9591501, complement
Nda2	XP_014502614.1	NC_028356.1	XM_014647128.2	LOC106762983	Vr6: 16917942..16922647, complement
Nda3	XP_014506992.1	NC_028357.1	XM_014651506.2	LOC106766760	Vr7: 36560587..36565425
Ndb1 X1	XP_014494071.1	NC_028352.1	XM_014638585.2	LOC106756246	Vr2: 10455058..10462038, complement

X2	XP_022634327.1	"	XM_022778606.1	"	"
X3	XP_022634328.1	"	XM_022778607.1	"	"
Ndb2	XP_014518088.1	NC_028360.1	XM_014662602.2	LOC106775476	Vr10: 19071744..19077301
Ndb3	XP_014506035.1	NC_028357.1	XM_014650549.2	LOC106765807	Vr7: 36369716..36374337, complement
Ndb4	XP_014493687.1	NC_028352.1	XM_014638201.2	LOC106755964	Vr2: 10449997..10454270, complement
Ndc1 X1	XP_014510386.1	NC_028357.1	XM_014654900.2	LOC106769327	Vr7: 55426185..55432695, complement
X2	XP_014510387.1	"	XM_014654901.2	"	"
Cajanus cajan					
Nda1 X1	XP_020237022.1	NW_017984249.1	XM_020381433.2	LOC109816420	Unplaced scaffold: 274478..279140, complement
X2	XP_020237023.1	"	XM_020381434.2	"	"
X3	XP_029130846.1	"	XM_029275013.1	"	"
Nda2 X1	XP_020202049.1	NW_017984470.1	XM_020346460.2	LOC109787872	Unplaced scaffold: 127649..133338, complement
X2	XP_020202050.1	"	XM_020346461.2	"	"
Nda3 X1	XP_020222667.1	NC_033812.1	XM_020367078.2	LOC109805107	Cc9: 7754001..7758452
X2	XP_020222668.1	"	XM_020367079.2	"	"
X3	XP_020222670.1	"	XM_020367081.2	"	"
X4	XP_020222671.1	"	XM_020367082.2	"	"
Ndb1	XP_020214940.1	NC_033807.1	XM_020359351.2	LOC109798916	Cc4: 9252591..9258024, complement
Ndb2	XP_020221799.1	NC_033811.1	XM_020366210.2	LOC109804393	Cc8: 18178398..18185223, complement
Ndb3	XP_020231485.1	NW_017984090.1	XM_020375896.2	LOC109812036	Unplaced scaffold: 172070..177654
Ndb4	XP_020215449.1	NC_033807.1	XM_020359860.2	LOC109799312	Cc4: 9245348..9251473, complement
Ndc1	XP_020203967.1	NW_017984654.1	XM_020348378.2	LOC109789425	Unplaced scaffold: 31672..37792, complement
Vigna unguiculata					
Nda1	XP_027933642.1	NC_040284.1	XM_028077841.1	LOC114189132	Vu6: 20802072..20805162, complement
Nda2	XP_027940962.1	NC_040286.1	XM_028085161.1	LOC114194763	Vu8: 28910164..28914467, complement
Nda3 X1	XP_027918804.1	NC_040281.1	XM_028063004.1	LOC114177594	Vu3: 10432557..10437629, complement
X2	XP_027918804.1	"	XM_028063003.1	"	"
Ndb1	XP_027911060.1	NC_040289.1	XM_028055259.1	LOC114169881	Vu11: 31125483..31131268
Ndb2	XP_027931187.1	NC_040284.1	XM_028075386.1	LOC114187206	Vu6: 32178971..32184437
Ndb3	XP_027920889.1	NC_040281.1	XM_028065088.1	LOC114178936	Vu3: 10616354..10621246
Ndb4	XP_027911308.1	NC_040289.1	XM_028055507.1	LOC114170040	Vu11: 31132429..31141245
Ndc1	XP_027915504.1	NC_040281.1	XM_028059703.1	LOC114174969	Vu3: 4891894..4898164, complement
Glycine soja					
Nda1.1	XP_028191813.1	NC_041012.1	XM_028336012.1	LOC114377485	Gs11: 24515480..24519819
Nda1.2 X1	XP_028212513.1	NC_041019.1	XM_028356712.1	LOC114395025	Gs18: 5689047..5693169
X2	XP_028212514.1	"	XM_028356713.1	"	"
Nda2.1 X1	XP_028214547.1	NC_041003.1	XM_028358746.1	LOC114396655	Gs2: 43609948..43615144, complement
X2	XP_028214554.1	"	XM_028358753.1	"	"
Nda2.2	XP_028199254.1	NC_041015.1	XM_028343453.1	LOC114383719	Gs14: 48439168..48444557, complement

Nda3	XP_028247057.1	NC_041009.1	XM_028391256.1	LOC114424415	Gs8: 4489345..4494589, complement
Ndb1	XP_028194852.1	NC_041013.1	XM_028339051.1	LOC114380115	Gs12: 9827554..9832771, complement
Ndb2	XP_028203628.1	NC_041016.1	XM_028347827.1	LOC114387624	Gs15: 5126036..5131167, complement
Ndb3	-	-	-	-	-
Ndb4.1	XP_028238195.1	NC_041007.1	XM_028382394.1	LOC114417256	Gs6: 46179209..46184762
Ndb4.2	XP_028194047.1	NC_041013.1	XM_028338246.1	LOC114379570	Gs12: 9819574..9824591, complement
Ndc1	XP_028233338.1	NC_041006.1	XM_028377537.1	LOC114413256	Gs5: 39875010..39880090
Arachis hypogaea					
Nda1	-	-	-	-	-
Nda2	XP_025609773.1	NC_037624.1	XM_025753988.2	-	Ah7: 22203099..22208123, complement
Nda3	-	-	-	-	-
Ndb1.1	XP_025687562.1	NC_037620.1	XM_025831777.2	-	Ah3: 19578286..19582707, complement
Ndb1.2 X1	XP_025614425.1	NC_037625.1	XM_025758640.2	-	Ah8: 33043674..33048418, complement
	XP_025614426.1	“	XM_025758641.2	-	“
	XP_025614427.1	“	XM_025758642.2	-	“
	XP_029144302.1	“	XM_029288469.1	-	“
	XP_029144303.1	“	XM_029288470.1	-	“
Ndb2	XP_025622092.1	NC_037627.1	XM_025766307.1	-	Ah10: 3189100..3193057
Ndb3	-	-	-	-	-
Ndb4.1	XP_029153212.1	NC_037620.1	XM_029297379.1	-	Ah3: 19573149..19577519, complement
Ndb4.2	XP_025637537.1	NC_037630.1	XM_025781752.2	-	Ah13: 20766901..20772467, complement
Ndc1 X1	XP_025689551.1	NC_037620.1	XM_025833766.2	-	Ah3: 119095018..119098745, complement
	XM_025833767.2	“	XP_025689552.1	-	“
Abrus precatorius					
Nda1 X1	XP_027335666.1	NW_020874439.1	XM_027479865.1	LOC113849739	Unplaced scaffold: 31027974..31031610, complement
X2	XP_027335665.1	“	XM_027479864.1	“	“
Nda2	XP_027369163.1	NW_020874428.1	XM_027513362.1	LOC113874990	Unplaced scaffold: 20652425..20657651, complement
Nda3	XP_027348706.1	NW_020874362.1	XM_027492905.1	LOC113860213	Unplaced scaffold: 34547091..34551846, complement
Ndb1.1	XP_027346646.1	NW_020874362.1	XM_027490845.1	LOC113858285	Unplaced scaffold: 25551249..25557266
	XP_027346647.1	“	XM_027490846.1	“	“
Ndb1.2	XP_027339746.1	NW_020874303.1	XM_027483945.1	LOC113853536	Unplaced scaffold: 24934908..24940074
Ndb2	XP_027338117.1	NW_020874292.1	XM_027482316.1	LOC113852058	Unplaced scaffold: 22473697..22478751, complement
Ndb3 X1	XP_027345265.1	NW_020874362.1	XM_027489464.1	LOC113857498	Unplaced scaffold: 34746978..34752512
X2	XP_027345266.1	“	XM_027489465.1	“	“
X3	XP_027345267.1	“	XM_027489466.1	“	“
Ndb4 X1	XP_027347667.1	NW_020874362.1	XM_027491866.1	LOC113859050	Unplaced scaffold: 25560563..25564975
X2	XP_027347668.1	“	XM_027491867.1	“	“
Ndc1 X1	XP_027341715.1	NW_020874303.1	XM_027485914.1	LOC113854729	Unplaced scaffold: 16968085..16972947
X2	XP_027341716.1	“	XM_027485915.1	“	“

X3	XP_027341717.1	"	XM_027485916.1	"	"
Prospiopis alba					
Nda1.1	XP_028754718.1	NW_021632674.1	XM_028898885.1	LOC114714174	Unplaced scaffold: 39515..43940, complement
Nda1.2	XP_028753074.1	NW_021632639.1	XM_028897241.1	LOC114712692	Unplaced scaffold: 141094..145517
Nda2	-	-	-	-	-
Nda3.1 X1	XP_028779616.1	NW_021637568.1	XM_028923783.1	LOC114735996	Unplaced scaffold: 15032..19974
X2	XP_028779617.1	"	XM_028923784.1	"	"
X3	XP_028779619.1	"	XM_028923786.1	"	"
Nda3.2 X1	XP_028755219.1	NW_021636190.1	XM_028899386.1	LOC114714635	Unplaced scaffold: 461905..466860, complement
X2	XP_028755220.1	"	XM_028899387.1	"	"
X3	XP_028755221.1	"	XM_028899388.1	"	"
Ndb1	XP_028808447.1	NW_021635967.1	XM_028952614.1	LOC114763018	Unplaced scaffold: 59732..65426, complement
Ndb2	XP_028788704.1	NW_021633704.1	XM_028932871.1	LOC114744702	Unplaced scaffold: 301552..306383
Ndb3 X1	XP_028806571.1	NW_021631975.1	XM_028950738.1	LOC114761356	Unplaced scaffold: 88265..111814, complement
X2	XP_028806639.1	"	XM_028950806.1	"	"
X3	XP_028806714.1	"	XM_028950881.1	"	"
Ndb4	XP_028808448.1	NW_021635967.1	XM_028952615.1	LOC114763019	Unplaced scaffold: 55448..59063, complement
Ndc1.1	XP_028786585.1	NW_021633428.1	XM_028930752.1	LOC114742508	Unplaced scaffold: 109609..114183
Ndc1.2	XP_028770379.1	NW_021632864.1	XM_028914546.1	LOC114727796	Unplaced scaffold: 450777..455408
Lotus japonicas					
LjNda1	-	-	-	Lj0g3v0132519.1	Lj0: 60481147..60486135
LjNda2	-	-	-	Lj2g3v2279580.1	Lj2: 33309635..33315049
LjNda3	-	-	-	Lj4g3v0447890.1	Lj4: 6025388..6031130
LjNdb1	-	-	-	Lj3g3v2873580.1	Lj3: 35376473..35382563
LjNdb2	-	-	-	Lj6g3v2083620.1	Lj6: 24587757..24592778
LjNdb3	-	-	-	Lj4g3v0445550.1	Lj4: 5883185..5888896 (complement)
LjNdb4	-	-	-	Lj3g3v2873590.1	Lj3: 35384268..35385400
LjNdc1	-	-	-	Lj4g3v2827910.1	Lj4: 37948896..37953135

Table S5: Stress-responsive (up-regulated) ND genes from other legumes based on publicly available transcriptomic datasets. *M. truncatula* data obtained through Genevestigator. *L. japonicus* data obtained through Lotus Base. *G. max* genes were also screened using Genevestigator, however there was either no response to abiotic stress experiments (*GmNDA1.1, 2.1, 2.2, GmNDB1.1, 3.1* and *GmNDC1*), or no available probeset IDS (*GmNDA1.2, 3.1, 3.2, GmNDB2.1, 2.2, 2.3, 3.2, 4.1, 4.2* and *4.3*).

Gene	Stress Treatment	Brief Experimental Details	Fold Changes and p values	Reference
M. truncatula				
MtNDA1	No response	-	-	-
MtNDA2	Drought	Jemmalong, shoot, 4 days drought, 3 reps	FC 2, p = 0.008	Zhang et al. 2014
MtNDA3	Salt	Jemmalong, radicle, 180mM NaCl for 6h, 24h, 48 h, 3 reps	FC 8.2, p = 0.021<br>FC 5.4, p <0.001<br>FC 6.14, p = 0.044	Li et al. 2009
	Drought	Jemmalong, root, 2d, 3 reps	FC 2.6, p = 0.003	Zhang et al. 2014
	Ozone	Jemmalong, leaf, 6d, 2 reps	FC 13.3, p = 0.001<br>FC 13.6 p = 0.012	Iyer et al. 2013
	Cold	Jemmalong and F83005.5, hypocotyl, 10 deg vs 20 deg, 2 reps	FC 2.4, p = 0.021<br>FC 2, p = 0.013	Unpublished
	Low phosphorous	Jemmalong, root, 0.02 mM vs 2 mM P for ~8 weeks, 3 reps	FC 2.2, p = 0.017	Hogekamp et al. 2011
MtNDB1	No response	-	-	-
MtNDB2	N.D.	-	-	-
MtNDB3	Ozone	Jemmalong, leaf, 6 d ozone, 2 reps	FC 2.3 p < 0.001	Iyer et al. 2013
MtNDB4	N.D.	-	-	-
MtNDC1	No response	-	-	-
**L. japonicus**				
LjNDA1	No response	-	-	-
LjNDA2	No response	-	-	-
LjNDA3	Drought	Gifu WT and glutamine synthetase knockout, shoot, 4 days drought, 2 reps	FC of relative expression value = 2 (WT) and 2.4 (KO)	Diaz et al. 2010 (Lotus Base)
LjNDB1	No response	-	-	-
LjNDB2	Drought	Gifu WT and glutamine synthetase knockout, shoot, 4 days drought, 2-3 reps	FC relative expression = 1.9 (WT), 4.5 (KO)	Diaz et al. 2010
	Salt	Gifu, shoot, 50mM NaCl for 28 days, added after 8 days growth, 7 reps	FC relative expression = 2.1	Sanchez et al. 2008
	Salt	Gifu, shoot, 75 mM NaCl for 28 days, added from germination, 4-7 reps	FC relative expression = 2.2	Sanchez et al. 2008
LjNDB3	N.D.	-	-	-
LjNDB4	No response	-	-	-
LjNDC1	No response	-	-	-

Table S6: Significant correlation coefficients between AP gene transcript levels and gas exchange measurements during salinity stress. Based on mean data for each cultivar and analyzed separately for each timepoint (T5, T9 and T15) using Spearman correlation analysis in SPSS (v25, IBM).

**Photosynthesis**	**AOX1 T5**	**AOX1 T9**	**NDB2 T9**	**NDB3 T15**	**NDC1 T15**
R^2	0.990(+)	0.886(+)	0.771(+)	0.771(+)	0.771 (+)
p	<0.001	0.019	0.072	0.072	0.111

Table S7: Primers used for qPCR analysis of CaND genes.

	F Primer	R Primer
CaNDA1	AAGCAAGGAGGCAAAAGGGG	TGTTCCTCCAGCTGATGACG
CaNDA2	GGTTCCATACGGATTGCTGGTA	ATCAATGCCGATCCTTCCAC
CaNDA3	GCTTTCTGAAAATCCAGGCACA	ATATCGCTCACGAACATCACTCAA
CaNDA4	GCTTTCTGAAAATCCAGGCACA	ATATCGCTCACAAACATCATTCAT
CaNDA5	CCAACCAGTTGACAAAGGTAGC	CATGCTTCCAAGGTGCTTGT
CaNDB1	ACGGGATCGGTTTGTTCTGT	CATGCCTCTTAGCCTGACCAA
CaNDB2	GCAAACAAGTAAGCTGGCGT	CTCAGATGCGGCTTGAATCTC
CaNDB3	ATGCAAGCAAACAAGTCAGC	TTGCCATTGCAGGACTCTGG
CaNDB4	ATGAAAGACATGGCCACTTTGC	GAGCAGCAACCTGAGCTGTT
CaNDC1	GGCCACACTGCTAGGAAGAT	GTAAGCCAGCTGATCCCCAC

References

Claros, M. G.; Vincens, P., Computational method to predict mitochondrially imported proteins and their targeting sequences. *European Journal of Biochemistry* **1996,** 241, (3), 779-786.

Clifton, R.; Lister, R.; Parker, K. L.; Sappl, P. G.; Elhafez, D.; Millar, A. H.; Day, D. A.; Whelan, J., Stress-induced co-expression of alternative respiratory chain components in *Arabidopsis thaliana*. *Plant Molecular Biology* **2005,** 58, (2), 193-212.

Emanuelsson, O.; Nielsen, H.; Von Heijne, G., ChloroP, a neural network-based method for predicting chloroplast transit peptides and their cleavage sites. *Protein Science* **1999,** 8, (5), 978-984.

Garg, R.; Patel, R. K.; Jhanwar, S.; Priya, P.; Bhattacharjee, A.; Yadav, G.; Bhatia, S.; Chattopadhyay, D.; Tyagi, A. K.; Jain, M., Gene discovery and tissue-specific transcriptome analysis in chickpea with massively parallel pyrosequencing and web resource development. *Plant Physiology* **2011,** 156, (4), 1661-1678.

Geisler, D. A.; Broselid, C.; Hederstedt, L.; Rasmusson, A. G., Ca^{2+}-binding and Ca^{2+}-independent respiratory NADH and NADPH dehydrogenases of *Arabidopsis thaliana*. *Journal of Biological Chemistry* **2007,** 282, (39), 28455-28464.

Hao, M. S.; Rasmusson, A. G., The evolution of substrate specificity-associated residues and Ca^{2+}-binding motifs in EF-hand-containing type II NAD(P)H dehydrogenases. *Physiologia Plantarum* **2016,** 157, (3), 338-351.

Lescot, M.; Dehais, P.; Thijs, G.; Marchal, K.; Moreau, Y.; Van de Peer, Y.; Rouze, P.; Rombauts, S., PlantCARE, a database of plant *cis*-acting regulatory elements and a portal to tools for in silico analysis of promoter sequences. *Nucleic Acids Research* **2002,** 30, (1), 325-327.

Michalecka, A. M.; Svensson, A. S.; Johansson, F. I.; Agius, S. C.; Johanson, U.; Brennicke, A.; Binder, S.; Rasmusson, A. G., Arabidopsis genes encoding mitochondrial type II NAD(P)H dehydrogenases have different evolutionary orgin and show distinct responses to light. *Plant Physiology* **2003,** 133, (2), 642-652.

Sanchez, D. H.; Lippold, F.; Redestig, H.; Hannah, M. A.; Erban, A.; Kramer, U.; Kopka, J.; Udvardi, M. K., Integrative functional genomics of salt acclimatization in the model legume *Lotus japonicus*. *Plant Journal* **2008,** 53, (6), 973-987.

Sanchez, D. H.; Pieckenstain, F. L.; Szymanski, J.; Erban, A.; Bromke, M.; Hannah, M. A.; Kraemer, U.; Kopka, J.; Udvardi, M. K., Comparative functional genomics of salt stress in related model and cultivated plants identifies and overcomes limitations to translational genomics. *Plos One* **2011,** 6, (2).

Small, I.; Peeters, N.; Legeai, F.; Lurin, C., Predotar: A tool for rapidly screening proteomes for N-terminal targeting sequences. *Proteomics* **2004,** 4, (6), 1581-1590.

Sweetman, C.; Khassanova, G.; Miller, T. K.; Booth, N. J.; Kurishbayev, A.; Jatayev, S.; Gupta, N. K.; Langridge, P.; Jenkins, C. L. D.; Soole, K. L.; Day, D. A.; Shavrukov, Y., Salt-induced expression of intracellular vesicle trafficking genes, CaRab-GTP, and their association with Na+ accumulation in leaves of chickpea (*Cicer arietinum* L.). *BMC Plant Biology* **In Press**.Thirkettle-Watts, D.; McCabe, T. C.; Clifton, R.; Moore, C.; Finnegan, P. M.; Day, D. A.; Whelan, J., Analysis of the alternative oxidase promoters from soybean. *Plant Physiology* **2003,** 133, (3), 1158-1169.
